# Human kallikrein gene 5 (KLK5) expression is an indicator of poor prognosis in ovarian cancer

**DOI:** 10.1054/bjoc.2000.1649

**Published:** 2001-03

**Authors:** H Kim, A Scorilas, D Katsaros, G M Yousef, M Massobrio, S Fracchioli, R Piccinno, G Gordini, E P Diamandis

**Affiliations:** Department of Pathology and Laboratory Medicine, Mount Sinai Hospital, 600 University Ave, Toronto, M5G 1X5, Canada; Department of Laboratory Medicine and Pathobiology, University of Toronto, 100 College Street, Toronto, Ontario, M5G 1L5, Canada; Department of Obstetrics and Gynecology, Gynecologic Oncology Unit, University of Turin, Turin, 10126, Italy; Department of Pathology, S. Anna Hospital, Turin, 10126, Italy

**Keywords:** kallikrein gene 5, ovarian carcinoma, gene expression, survival analysis, prognostic markers, human kallikreins

## Abstract

Kallikrein gene 5 (KLK5, also known as KLK-L2), located on chromosome 19q13.4, is one of the newly identified members of the kallikrein gene family, which is a subgroup of the serine protease enzyme family. In normal human tissues, KLK5 is highly expressed in skin, mammary gland and testis. Preliminary RT-PCR analysis has indicated that KLK5 is expressed in a subset of ovarian tumours. We have thus hypothesized that KLK5 may be a new prognostic indicator in ovarian cancer. We have examined the mRNA expression of KLK5 in 142 malignant ovarian tissues. Tumours were pulverized, total RNA was extracted, and cDNA was prepared by reverse transcription. KLK5 was amplified by PCR using gene specific primers, and the identity of the PCR product was verified by sequencing. Ovarian tissues were then classified as KLK5 positive or negative, based on ethidium bromide staining of the PCR product on agarose gels. KLK5 was found to be highly expressed in 58/142 (41%) of ovarian cancer samples while its level of expression was very low in normal ovarian tissues. We found a strong positive relation between KLK5 expression and tumour grade (*P* = 0.006) and disease stage (*P* = 0.027). Univariate survival analysis revealed that patients with ovarian tumours positive for KLK5 expression had an increased risk for relapse and death (*P* = 0.018 and 0.022, respectively). In multivariate analysis, KLK5 expression showed independent prognostic value only in the subset of tumours with lower grade disease (grades I and II). We conclude that KLK5 expression is associated with more aggressive forms of epithelial ovarian carcinoma and has indepdent prognostic value in low grade tumours. © 2001 Cancer Research Campaign http://www.bjcancer.com


*British Journal of Cancer* (2001) **84**(5), 643–650
© 2001 Cancer Research Campaign
doi: 10.1054/ bjoc.2000.1649, available online at http://www.idealibrary.com on IDEAL

http://www.bjcancer.com

### Human kallikrein gene 5 (KLK5) expression is an indicator of poor prognosis in ovarian cancer

**H Kim[1,2], A Scorilas[1,2], D Katsaros[3], GM Yousef[1,2], M Massobrio[3], S Fracchioli[3], R Piccinno[3], G Gordini[4] and EP Diamandis[1,2]**

[1]Department of Pathology and Laboratory Medicine, Mount Sinai Hospital, 600 University Ave, Toronto, Canada M5G 1X5; [2]Department of Laboratory Medicine and Pathobiology, University of Toronto, 100 College Street, Toronto, Ontario, Canada M5G 1L5; [3]Department of Obstetrics and Gynecology, Gynecologic Oncology Unit, University of Turin, Turin, Italy 10126; [4]Department of Pathology, S. Anna Hospital, Turin, Italy 10126

**Summary** Kallikrein gene 5 (KLK5, also known as KLK-L2), located on chromosome 19q13.4, is one of the newly identified members of the kallikrein gene family, which is a subgroup of the serine protease enzyme family. In normal human tissues, KLK5 is highly expressed in skin, mammary gland and testis. Preliminary RT-PCR analysis has indicated that KLK5 is expressed in a subset of ovarian tumours. We have thus hypothesized that KLK5 may be a new prognostic indicator in ovarian cancer. We have examined the mRNA expression of KLK5 in 142 malignant ovarian tissues. Tumours were pulverized, total RNA was extracted, and cDNA was prepared by reverse transcription. KLK5 was amplified by PCR using gene specific primers, and the identity of the PCR product was verified by sequencing. Ovarian tissues were then classified as KLK5 positive or negative, based on ethidium bromide staining of the PCR product on agarose gels. KLK5 was found to be highly expressed in 58/142 (41%) of ovarian cancer samples while its level of expression was very low in normal ovarian tissues. We found a strong positive relation between KLK5 expression and tumour grade ($P = 0.006$) and disease stage ($P = 0.027$). Univariate survival analysis revealed that patients with ovarian tumours positive for KLK5 expression had an increased risk for relapse and death ($P$ = 0.018 and 0.022, respectively). In multivariate analysis, KLK5 expression showed independent prognostic value only in the subset of tumours with lower grade disease (grades I and II). We conclude that KLK5 expression is associated with more aggressive forms of epithelial ovarian carcinoma and has indepdent prognostic value in low grade tumours. © 2001 Cancer Research Campaign http://www.bjcancer.com

**Keywords**: kallikrein gene 5; ovarian carcinoma; gene expression; survival analysis; prognostic markers; human kallikreins

Ovarian cancer is the second most common and the most lethal gynaecologic malignancy (Boring et al, 1994; England et al, 1995) with an average 5-year survival rate of 39% (Kristensen and Trope, 1997). The low survival rate is mainly due to the lack of sensitive tests for detection of early stage disease, which is often asymptomatic. At the time of diagnosis, about two thirds of the patients manifest advanced stage disease. Therefore, identification of reliable diagnostic biomarkers would greatly improve the outcomes of ovarian cancer by enabling early detection. Furthermore, identification of prognostic and predictive biomarkers would aid in the optimal management of these patients. Genes which display altered expression in cancer cells are generally considered as candidate tumour biomarkers and should be evaluated for possible correlation between their expression and patient prognosis. In this endeavour, serine proteases have received much attention in recent years.

Serine proteases serve several functions during tumour progression, including stimulating cellular growth, angiogenesis as well as degradation of extracellular matrix (Tryggvason et al, 1987; Duffy, 1991; Liotta et al, 1991). In particular, the last function is thought to be critical in conferring the invasiveness and metastatic potential of cancer cells (Hanahan and Weinberg, 2000). Indeed, this biological role of serine proteases seems to accord well with clinical reports which show that aberrant expression of serine proteases such as the plasminogen activator correlate positively with the invasiveness and metastasis, hence poor prognosis in different malignancies (Monsky et al, 1991; Powell et al, 1993; Herszenyi et al, 1999; Kuhn et al, 1999; Herszenyi et al, 2000).

The kallikrein gene family comprises at least 14 structurally related genes around chromosome 19q13.2-q13.4. (Yousef et al, 1999; Diamandis et al, 2000). Kallikrein gene 5 (KLK5; also known as KLK-L2 or human stratum corneum tryptic enzyme [HSCTE]) is one of the newly identified members of this gene family (Yousef and Diamandis, 1999). Structurally, KLK5 is likely to be synthesized as a preproenzyme that contains an N-terminal signal peptide (prezymogen) followed by an activation peptide and the enzymatic domain. In addition, sequence homology analysis relates KLK5 most closely with the enamel matrix serine proteinase 1 (EMSP1) while sharing a high degree of homology with other kallikreins, including PSA. Many new reports have shown that various members of the kallikrein gene family are differentially regulated in cancer. Specifically, the stratum corneum chymotryptic enzyme (KLK7), neuropsin (KLK8) and zyme (KLK6) have been shown to be overexpressed in ovarian tumours (Anisowicz et al, 1996; Tanimoto et al, 1999; Underwood et al, 1999) while the normal epithelial cell-specific 1 gene (NES1; KLK10) is down-regulated (Liu et al, 1996; Goyal et al, 1998).

*Received 25 September 2000*
*Revised 6 December 2000*
*Accepted 7 December 2000*

*Correspondence to:* EP Diamandis

In the present study, we have evaluated KLK5 mRNA expression in relation to patient survival and other clinicopathologic variables in epithelial ovarian carcinoma. Our results demonstrate that KLK5 is an independent prognostic marker of poor prognosis in a subset of patients with grade I/II tumours.

### MATERIALS AND METHODS

### Ovarian cancer specimens and patients

142 consecutive patients with epithelial ovarian carcinoma were included in this study, with ages ranging from 25 to 80 with a median of 59 years. Patients underwent surgery and treatment for primary ovarian carcinoma at the Department of Obstetrics and Gynecology, Gynecologic Oncology Unit, University of Turin, Turin, Italy between July, 1991 and April, 1999. Follow-up information (median follow-up period 48 months) was available for 138 patients, among whom 83 (60%) have relapsed and 55 (40%) died. Of the 142 ovarian adenocarcinomas, for which histological diagnosis was available, 64 (45%) were serous papillary, 26 (18%) endometrioid, 22 (16%) undifferentiated, 12 (8%) clear cell, 11 (8%) mucinous, 6 (4%) Mullerian and 1 (1%) was unclassified. Due to the relatively small sample size, the clear cell, mucinous and Mullerian types were combined into one category termed 'others'. Classification of histological types followed the World Health Organization criteria (Serov and Sorbin, 1973). All patients were staged according to the International Federation of Gynecology and Obstetrics staging system (Pettersson, 1994). Grading information was available for 141 patients, 10 (7%) had low potential malignancies (LPM), 17 (12%) had grade 1, 24 (17%) had grade 2 and 90 (64%) had grade 3 ovarian carcinoma. Grading was established for each ovarian tumour according to the criteria of Day et al. (1975). Debulked tumour specimens were stored in liquid nitrogen immediately after frozen section and shipped at −80˚C. All patients were treated with cisplatin-based chemotherapy regimens based on a platinum compound, alone or in association with other drugs; grade 1 and stage I patients received no further treatment. Investigations were performed in accordance with the Helsinki declaration and were approved by the Institute of Obstetrics and Gynecology, Turin, Italy.

### Preparation of total RNA

50–100 mg of tumour tissue was pulverized on dry ice, followed by total RNA extraction using the TRIzol® method (Gibco BRL, Gaithersburg, MD). Following measurement of total RNA concentration by spectrophotometry, 4 μg of total RNA from each tissue was used to carry out first strand cDNA synthesis using the SuperScript™ Preamplification System, as prescribed by the manufacturer (Gibco BRL). To test the success of cDNA synthesis, 1 μl of the reverse transcription product was amplified using PCR with primers specific for actin. Product was visualized on a 2% agarose gel stained with ethidium bromide.

### Assessment of KLK5 expression

One μl of the first strand cDNA product was amplified using HotStar Taq DNA polymerase in PCR reactions as prescribed by the manufacturer (Qiagen Inc, Mississauga, Ontario, Canada). Primers used in this reaction (5′CAA GAC CCC CCT GGA TGT GG3′ and 5′CCGAGACGGACTCTGAAA ACT TTCTTCC3′) were specific for KLK5. PCR reactions were carried out in an Eppendorf thermocycler (enzyme activation at 95˚C for 15 min; 40 cycles of denaturation at 94˚C for 30 s, annealing at 65˚C for 1 min, extension at 72˚C for 30 s; final extension at 72˚C for 10 min) and the 340 bp KLK5 PCR product was visualized with ethidium bromide on 2% agarose gels. Gels were photographed under UV light with Speedlight Gel Documentation System (Lightools Research, Encinitas, CA) using 20 × magnification. Images were scored by 2 independent observers for presence (positive) or absence (negative) of a KLK5 PCR-generated band. Each PCR reaction was performed twice to evaluate reproducibility of data.

### Statistical analysis

Associations between clinicopathological parameters such as stage, grade, histotype and residual tumour, and KLK5 expression were analysed by the chi-square test or the Fisher's Exact Test when appropriate.

For survival analysis, two different end points – cancer relapse (either local recurrence or distant metastasis) and death – were used to calculate progression free and overall survival, respectively. Progression-free survival was defined as the time interval between the date of surgery and the date of identification of recurrent or metastatic disease. Overall survival was defined as the time interval between the date of surgery and the date of death.

The Cox univariate and multivariate proportional hazard regression model (Cox, 1972) was used to evaluate the hazard ratio (relative risk of relapse or death in the KLK5-positive group). In the multivariate analysis, the models were adjusted for KLK5 expression, clinical stage, histologic grade, residual tumour and age.

Kaplan–Meier survival curves (Kaplan and Meier, 1958) were constructed for KLK5-positive and KLK5-negative patients. For further analysis, patients were divided into 2 groups either by the tumour grade (grade I–II vs. grade III) or by the success of debulking (optimal vs. suboptimal debulking group). In each category, survival rates (disease-free survival and overall survival) were compared between KLK5-positive and KLK5-negative groups. The differences between the survival curves between groups were tested for statistical significance by the log rank test (Mantel, 1966).

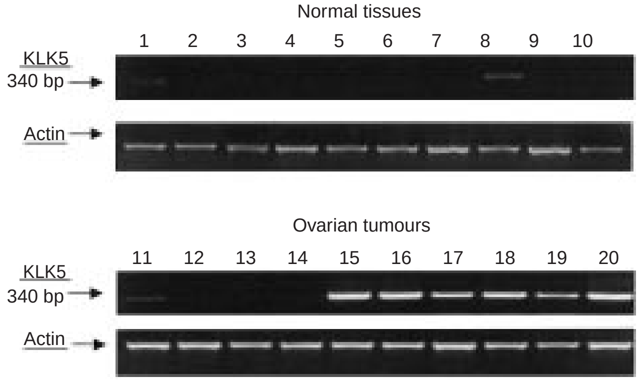

**Figure 1** Expression of the KLK5 gene as determined by RT-PCR. Total RNA was extracted from normal ($n$ = 10, top) and cancerous ovarian tissues ($n$ = 142, a representative blot shown in bottom), and used for RT-PCR. Actin was used as a control gene

© 2001 Cancer Research Campaign

**Table 1** Relationship between KLK5 status and other variables in primary ovarian cancer

| Variable | Patients | No. of patients (%) KLK5 negative | KLK5 positive | *P* value |
|---|---|---|---|---|
| Stage | | | | |
| I | 28 | 22 (78.6) | 6 (21.4) | |
| II | 10 | 7 (70.0) | 3 (30.0) | 0.027[a] |
| III | 92 | 50 (54.3) | 42 (45.7) | |
| IV | 10 | 3 (30.0) | 7 (70.0) | |
| X | 2 | | | |
| Grade | | | | |
| GB (Borderline) | 10 | 9 (90.1) | 1 (10.0) | |
| G1 | 17 | 15 (88.2) | 2 (11.8) | 0.006[a] |
| G2 | 24 | 13 (54.2) | 11 (45.8) | |
| G3 | 90 | 46 (51.1) | 44 (48.9) | |
| X | 1 | | | |
| Histotype | | | | |
| Serous | 64 | 36 (56.3) | 28 (43.8) | |
| Endometrioid | 26 | 17 (65.4) | 9 (34.6) | 0.789[a] |
| Undifferentiated | 22 | 12 (54.5) | 10 (45.5) | |
| Others | 30 | 19 (63.3) | 11 (36.7) | |
| Residual tumor (cm) | | | | |
| 0 | 57 | 39 (68.4) | 18 (31.6) | |
| 1–2 | 27 | 12 (44.4) | 15 (55.6) | 0.103[a] |
| >2 | 56 | 32 (57.1) | 24 (42.9) | |
| X | 2 | | | |
| Debulking success[c] | | | | |
| OD | 69 | 46 (66.7) | 23 (33.3) | 0.088[b] |
| SO | 71 | 37 (52.1) | 34 (47.9) | |
| X | 2 | | | |
| Menopause | | | | |
| Pre/peri | 44 | 22 (50.0) | 22 (50.0) | 0.145[b] |
| Post | 98 | 62 (63.3) | 36 (36.7) | |
| Response to CTX[d] | | | | |
| NC/PD | 16 | 5 (31.3) | 11 (68.8) | 0.029[b] |
| CR/PR | 121 | 75 (62.0) | 46 (38.0) | |
| NE | 5 | | | |

[a]$\chi^2$ test; [b]Fisher's Exact Test; [c]OD; optimal debulking (0–1 cm), SO; suboptimal debulking (>1 cm); [d]CTX; chemotherapy, NC; no change, PD; progressive disease, CR; complete response, PR; partial response, NE; not evaluated; X; status unknown.

### RESULTS

### Relationship between KLK5 expression and other parameters

From the 142 patients included in this study, 58 (41%) were positive for KLK5 expression in their ovarian tissue. In 10 normal ovarian tissues, the level of KLK5 was undetectable or low (Figure 1). Table 1 depicts the distribution of KLK5 expression (positive or negative) in ovarian tumour tissues in relation to clinical stage, histological grade, histotype, size of residual tumour, menopausal status and chemotherapy response. A significantly higher percentage of patients with advanced stage ($P = 0.027$) and higher grade ($P = 0.006$) tumour had detectable KLK5 expression. On the other hand, comparison of KLK5 expression in relation to response to chemotherapy revealed that KLK5 positivity was associated with lack of response ($P = 0.029$). No significant associations were found between KLK5 expression and different histotypes, residual tumour size, debulking success or menopausal status.

### Survival analysis

Out of the 142 patients included in this study, follow-up information was available for 138 of them with a median follow-up period of 48 months. 83 patients suffered a relapse and 55 of them died. Table 2 presents the association between cancer relapse or death, and various prognostic parameters. Data from the univariate analysis suggest that patients positive for KLK5 expression have 1.68- and 1.83-fold greater risk of relapse ($P = 0.018$) and death ($P = 0.022$), respectively. As expected, disease stage, grading and residual tumour were found to be strongly associated with increased risks of relapse and death ($P < 0.001$). Kaplan–Meier survival curves (Figure 2) demonstrate that KLK5-negative patients live longer and relapse less frequently. On the other hand, when Cox analysis was adjusted by stage of disease, grading, residual tumour, histologic type and age in the multivariate model, the prognostic value of KLK5 was no longer statistically significant. As expected, disease stage and residual tumour size were found to be the strongest independent indicators of poor prognosis.

© 2001 Cancer Research Campaign

**Table 2** Univariate and multivariate analysis of KLK5 with regard to progression-free (PFS) and overall (OS) survival

| Variable | Progression-free survival | | | Overall survival | | |
|---|---|---|---|---|---|---|
| | HR[a] | 95% CI[b] | *P* value | HR[a] | 95% CI[b] | *P* value |
| *Univariate analysis* | | | | | | |
| KLK5 | | | | | | |
| Negative | 1.00 | | | 1.00 | | |
| Positive | 1.68 | 1.09–2.59 | 0.018 | 1.83 | 1.09–3.06 | 0.022 |
| Stage of disease (ordinal) | 2.96 | 2.08–4.21 | <0.001 | 3.52 | 2.25–5.52 | <0.001 |
| Grading (ordinal) | 2.26 | 1.60–3.21 | <0.001 | 2.55 | 1.59–4.07 | <0.001 |
| Residual tumour (ordinal) | 1.25 | 1.17–1.32 | <0.001 | 1.31 | 1.21–1.41 | <0.001 |
| Histologic type[c] | 1.42 | 0.92–2.19 | 0.11 | 1.33 | 0.79–2.24 | 0.27 |
| Age | 1.01 | 0.99–1.03 | 0.18 | 1.01 | 0.99–1.03 | 0.21 |
| *Multivariate analysis* | | | | | | |
| KLK5 | | | | | | |
| Negative | 1.00 | | | 1.00 | | |
| Positive | 1.27 | 0.78–2.06 | 0.32 | 1.45 | 0.82–2.54 | 0.19 |
| Stage of disease (ordinal) | 1.87 | 1.28–2.75 | 0.001 | 2.03 | 1.24–3.31 | 0.004 |
| Grading (ordinal) | 1.45 | 0.97–2.18 | 0.061 | 1.55 | 0.90–2.69 | 0.11 |
| Residual tumour (ordinal) | 1.13 | 1.05–1.22 | 0.001 | 1.21 | 1.10–1.32 | <0.001 |
| Histologic type[c] | 1.06 | 0.67–1.68 | 0.78 | 0.83 | 0.48–1.43 | 0.51 |
| Age | 1.02 | 0.99–1.04 | 0.12 | 1.02 | 0.99–1.05 | 0.095 |

[a]Hazard ratio (HR) estimated from Cox proportional hazard regression model; [b]Confidence interval of the estimated HR; [c]Endometrioid, undifferentiated and others vs. serous.

### Survival analysis in patients stratified by the tumour stage, grade or success of debulking

We further examined the associations between the presence of KLK5 and cancer relapse or death in subgroups of patients who were categorized based on either stage (stage I and II vs III and IV) histologic grade (grade I/II vs. grade III) or debulking success (optimal debulking vs. suboptimal debulking). Among patients with grade I/II tumours, KLK5 positivity was associated with approximately 3–4-fold greater risk of relapse in both univariate ($P = 0.006$) and multivariate analysis ($P = 0.018$) (Table 3). Similarly, there was a tendency for increased risk of death in the KLK5-positive group, but this did not reach statistical significance in the Cox regression model ($P = 0.055$). On the other hand, Kaplan–Meier curves (Figure 3) demonstrate that among patients with grade I/II tumours, KLK5-negative patients show significantly higher probability of both relapse-free survival ($P = 0.003$) and overall survival ($P = 0.043$). Conversely, as shown in Table 3 and Figure 4, patients with grade III tumours were found to be equally likely to suffer a relapse or die, regardless of their KLK5 expression status, with comparable hazard ratios between KLK5-positive and -negative groups.

Table 4 presents relative risks of relapse and death of KLK-positive or -negative patients, in relation to their debulking success. Among patients showing optimal debulking, presence of KLK5 was associated with an approximately 3-fold increase in the risk of relapse in both univariate ($P = 0.008$) and multivariate analysis ($P = 0.049$) while KLK5 expression status did not show a significant relation with the risk of death. This finding was consistent with the results of the Kaplan–Meier survival analysis (Figure 5). In the group of patients with suboptimal debulking, KLK5 expression was associated with an increased (two-fold) risk of death in both univariate ($P = 0.023$) and multivariate analysis ($P = 0.006$) while no significant association was found between the KLK5 expression and the risk of relapse (Table 4). Kaplan–Meier survival curves produced similar data (Figure 6).

No statistically significant association was found between KLK5 expression and risk of relapse or death in the group of patients with stage I and II vs III and IV, in both univariate and multivariate analysis. Kaplan–Meier survival curves produced similar results (data not shown).

### DISCUSSION

Protease-mediated degradation of extracellular matrix promotes tumour invasiveness and metastasis. The present study demonstrates that KLK5 expression is low in normal ovaries but it is significantly higher in a subset (~40%) of ovarian carcinomas. Higher expression of KLK5 was found to be significantly more frequent in patients with advanced stage diseases and in poorly differentiated tumours (grade III). The present study does not provide any evidence for a direct cause and effect relationship between KLK5 expression and tumour progression. However, it is reasonable to hypothesize a potential role of KLK5 in promoting tumour invasiveness and metastasis. As mentioned earlier, KLK5 structurally resembles EMSP-1 (54% amino acid sequence identity, 68% similarity), which degrades the enamel matrix protein and other serine proteinases (Diamandis et al, 2000 ). It would be interesting to investigate whether KLK5 is similar to EMSP-1 in terms of its enzymatic activity and substrates.

The observed overexpression of KLK5 mRNA in ovarian carcinoma suggests that KLK5 gene transcription may be enhanced in ovarian tumours. Our laboratory previously demonstrated that KLK5 is up-regulated by oestrogen (Yousef and Diamandis, 1999). It was recently found that during ovarian carcinogenesis, oestrogen receptor-α is dramatically increased (Pujol et al, 1998). Hence, it is conceivable that overexpression of KLK5 occurs as a

 © 2001 Cancer Research Campaign

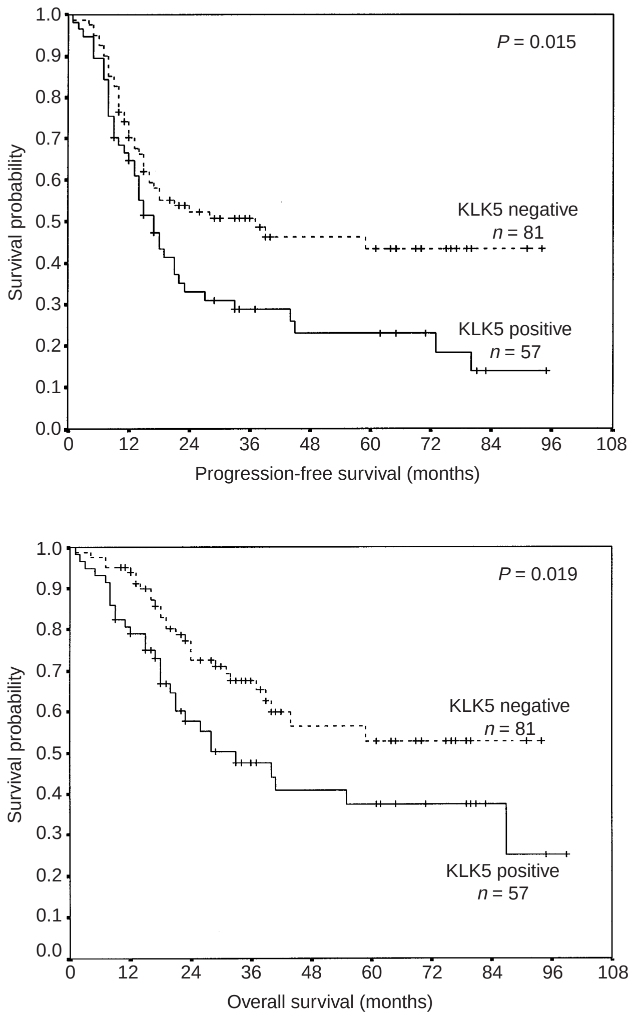

**Figure 2** Kaplan–Meier survival analysis of progression free (top) and overall survival (bottom) in patients with ovarian cancer who were either KLK5 positive or KLK5 negative. *n*: number of patients

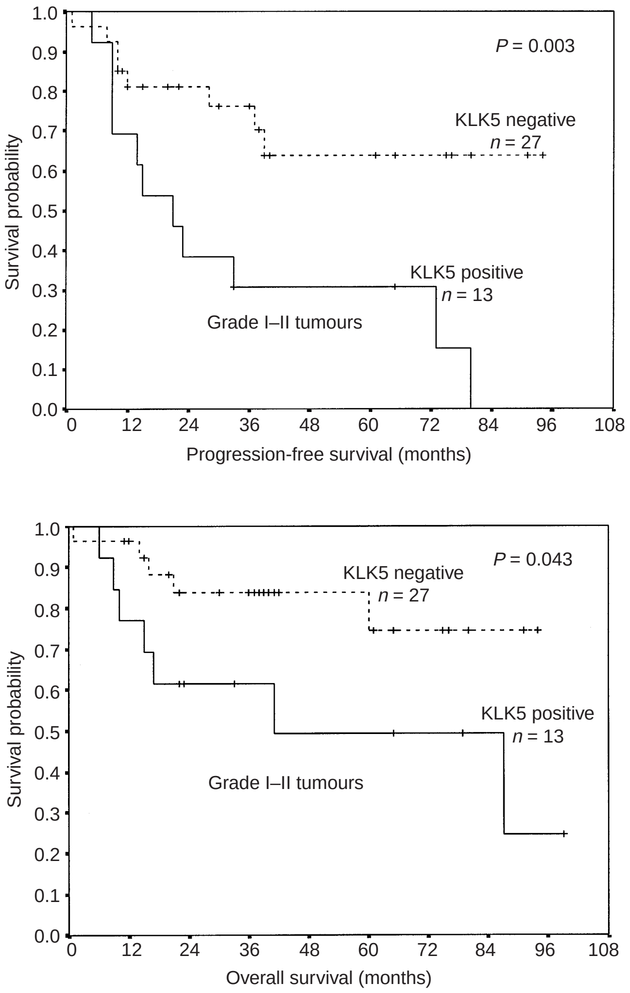

**Figure 3** Kaplan–Meier survival analysis of progression free (top) and overall survival (bottom) in patients with grade I/II tumour who were either KLK5 positive or KLK5 negative. *n*: number of patients

**Table 3** Associations between KLK5 and progression-free (PFS) and overall (OS) survival stratified by the tumour grade

| Variable | Progression-free survival | | | Overall survival | | |
|---|---|---|---|---|---|---|
| | **HR**[a] | **95% CI**[b] | ***P* value** | **HR**[a] | **95% CI**[b] | ***P* value** |
| **Tumour grade I–II** | | | | | | |
| *Univariate analysis* | | | | | | |
| KLK5 negative | 1.00 | | | 1.00 | | |
| KLK5 positive | 3.59 | 1.43–9.01 | 0.006 | 3.07 | 0.97–9.71 | 0.055 |
| **Multivariate analysis**[c] | | | | | | |
| KLK5 negative | 1.00 | | | 1.00 | | |
| KLK5 positive | 3.89 | 1.26–12.02 | 0.018 | 3.26 | 0.74–14.23 | 0.12 |
| **Tumour grade III** | | | | | | |
| *Univariate analysis* | | | | | | |
| KLK5 negative | 1.00 | | | 1.00 | | |
| KLK5 positive | 1.07 | 0.65–1.75 | 0.78 | 1.37 | 0.76–2.44 | 0.29 |
| **Multivariate analysis**[c] | | | | | | |
| KLK5 negative | 1.00 | | | 1.00 | | |
| KLK5 positive | 1.19 | 0.67–2.11 | 0.56 | 1.51 | 0.76–2.96 | 0.23 |

[a]Hazard ratio (HR) estimated from Cox proportional hazard regression model; [b]Confidence interval of the estimated HR; [c]Multivariate models were adjusted for stage of disease, residual tumour, histologic type and age.

**Table 4** Associations between KLK5 and progression-free (PFS) and overall (OS) stratified by the debulking success

| Variable | Progression-free survival | | | Overall survival | | |
|---|---|---|---|---|---|---|
| | HR[a] | 95% CI[b] | *P* value | HR[a] | 95% CI[b] | *P* value |
| **Optimal debulking** | | | | | | |
| *Univariate analysis* | | | | | | |
| KLK5 negative | 1.00 | | | 1.00 | | |
| KLK5 positive | 3.08 | 1.33–7.13 | 0.008 | 1.83 | 0.59–5.70 | 0.29 |
| *Multivariate analysis*[c] | | | | | | |
| KLK5 negative | 1.00 | | | 1.00 | | |
| KLK5 positive | 2.86 | 1.02–8.06 | 0.049 | 1.14 | 0.33–3.97 | 0.83 |
| **Suboptimal debulking** | | | | | | |
| *Univariate analysis* | | | | | | |
| KLK5 negative | 1.00 | | | 1.00 | | |
| KLK5 positive | 1.23 | 0.74–2.03 | 0.42 | 1.95 | 1.09–3.47 | 0.023 |
| *Multivariate analysis*[c] | | | | | | |
| KLK5 negative | 1.00 | | | 1.00 | | |
| KLK5 positive | 1.27 | 0.68–2.36 | 0.44 | 2.56 | 1.31–5.03 | 0.006 |

[a]Hazard ratio (HR) estimated from Cox proportional hazard regression model; [b]Confidence interval of the estimated HR; [c]Multivariate models were adjusted for stage of disease, tumour grade, histologic type and age.

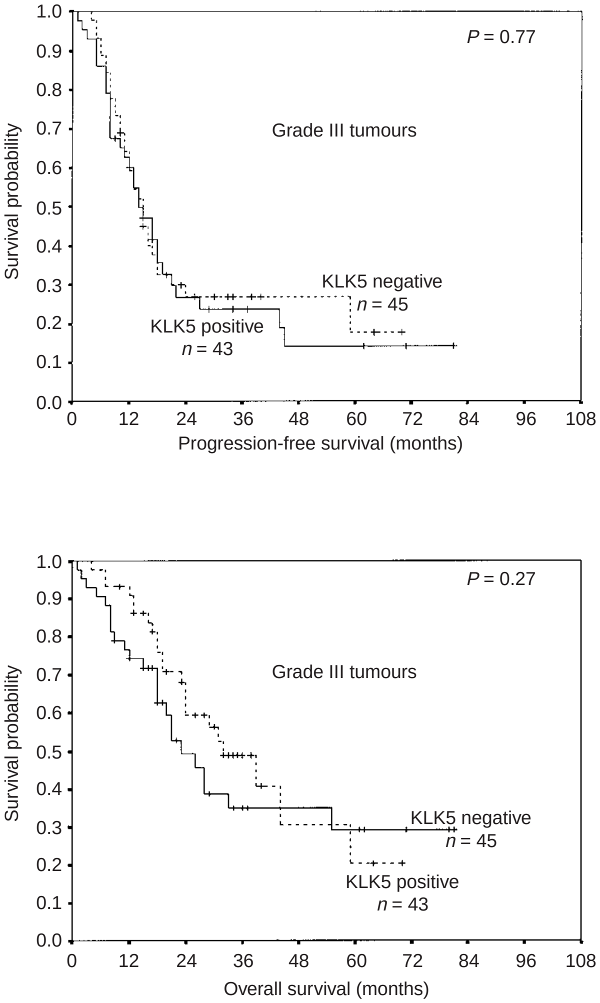

**Figure 4** Kaplan–Meier survival analysis of progression free (top) and overall survival (bottom) in patients with grade III tumour who were either KLK5 positive or KLK5 negative. *n*: number of patients

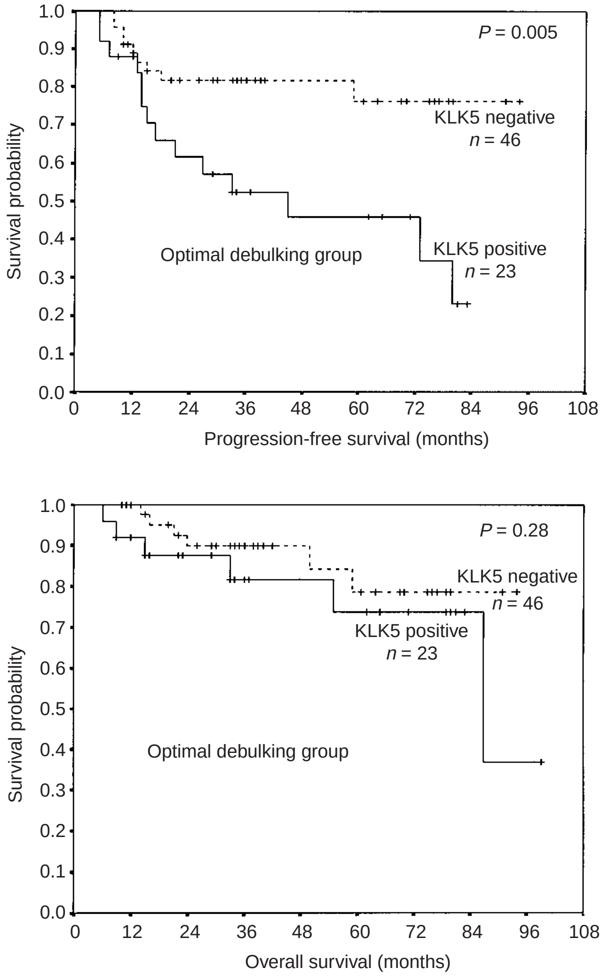

**Figure 5** Kaplan–Meier survival analysis of progression free (top) and overall survival (bottom) in patients with optimal debulking who were either KLK5 positive or KLK5 negative. *n*: number of patients

© 2001 Cancer Research Campaign

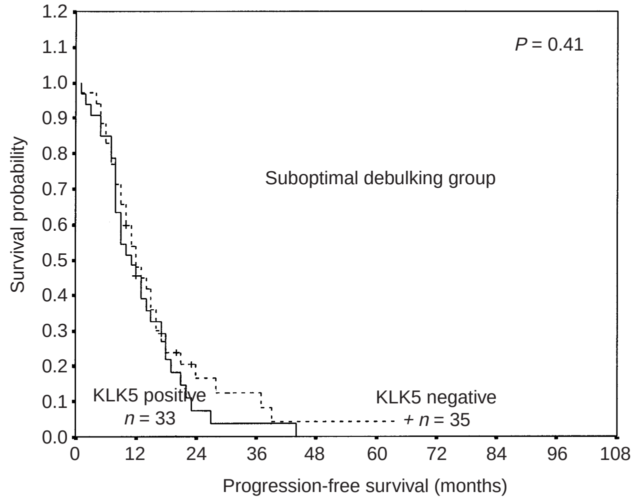

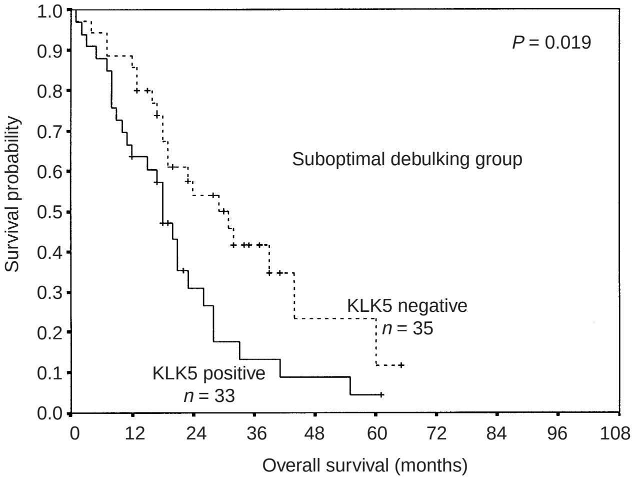

**Figure 6** Kaplan–Meier survival analysis of progression free (top) and overall survival (bottom) in patients with suboptimal debulking who were either KLK5 positive or KLK5 negative. *n*: number of patients result of an increased number of hormone-receptor complexes, which would, in turn, up-regulate KLK5. Further investigation is warranted to address this question.

Loss of differentiation in higher-grade tumours occurs as a result of altered cellular signalling pathways which govern cell growth and differentiation (reviewed in Hanahan and Weinberg, 2000). In essence, changes of cell signalling in cancer cells occur in favour of growth while circumventing terminal differentiation. As shown in Table 1, KLK5 expression increases most noticeably between grade I and grade II tumours, raising the question of a potential link between KLK5 and cellular differentiation or signalling pathways during tumour progression.

Our findings demonstrate that the KLK5 expression correlates with increased risks of relapse and death in univariate but not multivariate analysis (Table 2). This implies that KLK5 positivity is not an independent prognostic factor, but rather, a parameter associated with late-stage, and high-grade ovarian tumours. In early grade cancer, KLK5 expression does have independent prognostic value for a subgroup of patients (40 patients) but only for disease-free survival (Table 3). Among optimally debulked patients, KLK5 expression independently predicts disease-free survival while in suboptimally debulked patients, KLK5 expression independently predicts overall survival. Our lack of knowledge of KLK5 function and regulation in normal and cancerous tissues does not allow us to formulate reasonable hypotheses to explain these phenomenological observations. More studies with a larger group of patients will be necessary to substantiate these data.

In conclusion, the present study suggests that KLK5 could be a useful, independent prognostic indicator in patients with grade I/II ovarian tumours. In these patients, assessment of KLK5 expression could assist physicians to determine those who are at higher risk for relapse. While the presented data pertain to KLK5 mRNA expression, KLK5 protein is likely secreted (Yousef and Diamandis, 1999). Thus, the current findings provide evidence to support a potential utility of this gene and the encoded protein in developing a diagnostic test for ovarian cancer patients.

### REFERENCES

Anisowicz A, Sotiropoulou G, Stenman G, Mok SC and Sager R (1996) A novel protease homolog differentially expressed in breast and ovarian cancer. *Mol Med* **2**: 624–636

Boring CC, Squires TS, Tong T and Montgomery S (1994) Cancer statistics. *CA Cancer J Clin* **44**: 7–26

Cox DR (1972) Regression models and life tables. *J R Stat Soc (B)* **34**: 187–202

Diamandis E, Yousef G, Luo L-Y, Maklara A and Obiezu C (2000) The new human kallikrein gene family: implications in carcinogenesis. *Trends Endocrinol Metab* **11**: 54–60

Duffy MG (1991) The role of proteolytic enzymes in cancer invasion and metastasis. *Clin Exp Metastasis* **10**: 145–155

England A, Haldorsen T and Tretli S (1995) Prediction of cancer mortality in the Nordic countries up to the years 2000 and 2010 on the basis of relative survival analysis: a collaborative study of the five Nordic cancer registries. *APMIS Suppl* **49**: 81–83

Gallanger TGD Jr. and Rutledge FN (1975) Epithelial carcinoma of the ovary: prognostic importance of histologic grade. *Natl Cancer Inst Monogr* **42**: 15–21

Hanahan D and Weinberg RA (2000) The hallmarks of cancer. *Cell* **100**: 57–70

Herszenyi L, Plebani M, Carraro P, Paoli MD, Roveroni G, Cardin R, Tulassay Z, Naccarato R and Farinati F (1999) The role of cysteine and serine proteases in colorectal carcinoma. *Cancer* **86**: 1135–1142

Herszenyi L, Plebani M, Carraro P, Paoli MD, Roveroni G, Cardin R, Foschia F, Tulassay Z, Naccarato R and Farinati F (2000) Proteases in gastrointestinal neoplastic diseases. *Clin Chim Acta* **291**: 171–187

Kaplan EL and Meier P (1958) Nonparametric estimation from incomplete observations. *J Am Stat Assoc* **53**: 457–481

Kristensen GB and Trope C (1997) Epithelial ovarian carcinoma. *Lancet* **349**: 113–117

Kuhn W, Schmalfeldt B, Reuning U, Pache L, Berger U, Ulm K, Harbeck N, Spathe K, Dettmar P, Hofler H, Janicke F, Schmitt M and Graeft H (1999) Prognostic significance of urokinase (uPA) and its inhibitor PAI-1 for survival in advanced ovarian carcinoma stage FIGO IIIc. *Br J Cancer* **79**: 1746–1751

Liotta LA, Steeg PS and Stetler-Stevenson WG (1991) Cancer metastasis and angiogenesis: an imbalance of positive and negative regulation. *Cell* **64**: 327–336

Mantel N (1966) Evaluation of survival data and two new rank order statistics arising in its consideration. *Cancer Chemother Rep* **50**: 163–170

Monsky WL, Kelly T, Lin CY and Yeh Y (1991) Binding and localization of $M_r$ 72,000 matrix metalloproteinase at cell surface invadapodia. *Cancer Res* **53**: 3159–3164

Powell WC, Knox JD, Navre M, Grogan TM, Kittelson J, Nagel RB and Bowden GT (1993) Expression of the metalloproteinase Matrilysin in DU145 cells increases their invasive potential in severe combined immunodeficient mice. *Cancer Res* **53**: 417–422

Pujol P, Rey JM, Nirde P, Roger P, Gastaldi M, Laffargne F, Rochefort H and Mandelonde T (1998) Differential expression of estrogen receptor-α and β messenger RNAs as a potential marker of ovarian carcinogenesis. *Cancer Res* **58**: 5367–5373

© 2001 Cancer Research Campaign 

Serov SF and Sorbin LH (1973) *Histological typing of ovarian tumors. No. 9*. Geneva: World Health Organization 17

Tanimoto H, Underwood LJ, Shigemasa K, Yan MS, Clarke J, Parmley TH and O'Brien TJ (1999) The stratum corneum chymotryptic enzyme that mediates shedding and desquamation of skin cells is highly overexpressed in ovarian tumor cells. *Cancer* **86**: 2074–2082

Tryggvason K, Hoyhtya M and Salo T (1987) Proteolytic degradation of extracellular matrix in tumor invasion. *Biochim Biophys Acta* **907**: 191–217

Underwood LJ, Tanimoto H, Wang Y, Shigemasa K, Parmley TH and O'Brien TJ (1999) Cloning of tumor-associated differentially expressed gene-14, a novel serine protease overexpressed by ovarian carcinoma. *Cancer Res* **59**: 1999

Yousef GM and Diamandis EP (1999) The new kallikrein-like gene KLK-L2: molecular characterization, mapping, tissue expression and hormonal regulation. *J Biol Chem* **274**: 37511–37516

Yousef GM, Luo LY and Diamandis EP (1999) Identification of novel human kallikrein like genes on chromosome 19q13.3-q13.4. *Anticancer Res* **19**: 2843–2852

*© 2001 Cancer Research Campaign*
